# 

**Published:** 1864-05

**Authors:** 


AMERICAN MEDICAL ASSOCIATION.—The Fifteenth Annual Meeting of the "American Medical Association" will be held in the City of New York, commencing Tuesday, June 7th, 1864, at 10 A. M.

GUIDO FURMAN, M. D., Secretary.

New York, March, 1864.

### RECEIVED.

*The Diseases of the Ear, their Diagnosis and Treatment. A Text Book of Aural Surgery in the form of Academical Lectures. By Dr.* Anton Von Tröltsch, *Aural Surgeon and Lecturer in the University, in Wursburg, Bavaria. Translated from the German and edited by* D. B. St. John Rosa, M. D., *Assistant Surgeon to the New York Eye Infirmary. Illustrated with wood engravings. From the second and last German edition. New York:* Wm. Wood & Co., 1864.

*Defence of Brigadier-General* William A. Hammond, *Surgeon-General U. S. Army.*

*Laryngoscopal Medication; or, the Local Treatment of Diseases of the Throat, Larynx, and neighboring organs, under sight. By* Louis Elsberg, A. M., M. D., *Lecturer on the Diseases of the Larynx and Throat in the University of New York. From Papers read before the American Medical Association, New York Academy of Medicine, and New York County Medical Society, and published in the Transactions of these Societies. With seven wood cuts representing the various Implements employed. New York:* Wm. Wood & Co., 1864.

*United States Sanitary Commission, No.* 79. *Department of the Special Inspection of the General Hospitals U. S. Army. Third (preliminary) Report of the Committee, May,* 1863. *By* Henry G. Clark, M. D., *Inspector-in-Chief.*

*Twenty-Fifth Annual Report of the Board of Trustees and Officers of the Central Ohio Lunatic Asylum, to the Governor of the State of Ohio, for the year* 1863.

